# Advances in 3D Printing Applications for Personalized Orthopedic Surgery: From Anatomical Modeling to Patient-Specific Implants

**DOI:** 10.3390/jcm14113989

**Published:** 2025-06-05

**Authors:** Marcin Prządka, Weronika Pająk, Jakub Kleinrok, Joanna Pec, Karolina Michno, Robert Karpiński, Jacek Baj

**Affiliations:** 1 Department of Orthopedics and Movement Traumatology, Provincial Integrated Hospital, Szpitalna 45, 62-504 Konin, Poland; marcinprzadka@poczta.onet.pl; 2 Department of Correct, Clinical and Imaging Anatomy, Medical University of Lublin, ul. Jaczewskiego 4, 20-090 Lublin, Poland; wapajak@gmail.com (W.P.); apec1410@gmail.com (J.P.); 3 Department of Forensic Medicine, Medical University of Lublin, Jaczewskiego 8b, 20-090 Lublin, Poland; klejs.90@gmail.com (J.K.); karolina.michno@stud.umed.lodz.pl (K.M.); 4 Department of Machine Design and Mechatronics, Faculty of 1 Mechanical Engineering, Lublin University of Technology, Nadbystrzycka 36, 20-618 Lublin, Poland; 5 Institute of Medical Sciences, The John Paul II Catholic University of Lublin, ul. Konstantynów 1H, 20-708 Lublin, Poland

**Keywords:** orthopedic implants, biomaterials, biocompatibility, tissue engineering

## Abstract

Three-dimensional (3D) printing has gained substantial interest among scientists and surgeons over the past decade due to its broad potential in medical applications. Its clinical utility has been increasingly recognized, demonstrating promising outcomes for patient care. Currently, 3D printing technology enables surgeons to enhance operative precision by facilitating the creation of patient-specific anatomical models, customized implants, biological tissues, and even surgical instruments. This personalization contributes to improved surgical outcomes, reduced operative times, and shorter postoperative recovery periods. Furthermore, 3D printing significantly aids in the customization of prostheses to conform closely to individual anatomical structures. Beyond therapeutic applications, 3D printing serves as a valuable educational tool in medical training. It enhances case-specific visualization, elucidates fracture mechanisms, and provides tangible models for simulation-based practice. Although the use of 3D printing might be seen as useful mostly in orthopedics, it has expanded into multiple medical specialties, including plastic surgery, dentistry, and emergency medicine. Presently, 3D-printed constructs are routinely employed for preoperative planning, prosthetic development, fracture management, and the fabrication of patient-specific surgical tools. Futuristically, the integration of 3D printing into clinical practice is expected to play a pivotal role in the advancement of personalized medicine, offering substantial benefits for both healthcare providers and patients.

## 1. Introduction

**Figure 1:**
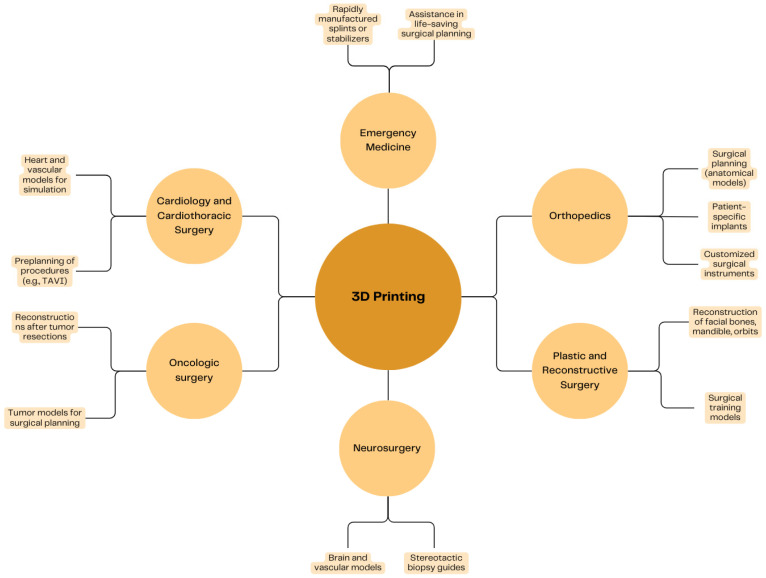
Applications of 3D printing (image prepared on the basis of analyzed data).

Over the past decade, 3D printing has provided significant advancement in orthopedic surgeries and trauma care, starting with basic prosthetics and progressing to advanced parts that are currently used, modern, and personally suited in shape and tissue. It is often referred to as ‘rapid prototyping (RP)’ or ‘additive manufacturing (AM)’ with its promising use for enhancing the precision of orthopedic surgeries [1]. Conventionally, orthopedic prosthetics were manufactured to be applied to various anatomical structures and tissue physiology. It would lead to different recovery problems, including longer rehabilitation. Therefore, introducing 3D printing technology was a remarkable breakthrough in many medical fields [2]. Three-dimensional printing technology offers unique flexibility in creating customized objects, which has led to growing interest among both surgeons and researchers. Unlike traditional manufacturing methods that excise material, 3D printing is an additive process, building objects layer by layer using substances like plastic or metal based on digital designs. In surgical settings, this technique can be used not only to create personalized anatomical models but also to produce personalized surgical tools, known as patient-specific instruments (PSIs). Moreover, this method is helpful with custom-made implants designed to meet the precise needs of each surgical case [3]. With the help of computer tomography (CT) and magnetic resonance (MR), surgeons can manufacture individually generated, anatomically and pathophysiologically suited models. In addition to orthopedic surgeries, 3D printing has been found to be useful in various medical fields, such as dentistry, plastic surgery, and neurosurgery [4]. In a futuristic approach, 3D printing may also be used as a teaching method for better understanding and treating orthopedic and traumatic cases [5]. The integration of 3D printing technology into orthopedic education significantly enhances the teaching and learning experience, particularly when combined with case-based learning (CBL). Given that orthopedic diagnoses and treatments are closely tied to complex anatomical structures. 3D-printed models offer a substantial, interactive representation of joint structures, allowing students to visualize and manipulate anatomical parts, which deepens their understanding of theoretical concepts such as fracture patterns and degenerative changes. Furthermore, by simulating surgical scenarios and preoperative planning, 3D printing supports the development of practical skills that are crucial for future orthopedic surgeons [6]. Orthopedic implants are commonly made from metals due to their strength and ability to support early patient mobility. However, traditional metal implant fabrication methods often struggle to meet personalized patient needs. Additionally, a mismatch in stiffness between metal implants and bone can cause stress shielding, which may lead to bone loss and implant failure [7]. Three-dimensional printing uses materials like powdered metal and plastic to create custom objects from digital models, with growing applications in orthopedics. Advanced materials and integration with fields like digital medicine are helping to produce personalized implants that enhance durability and patient satisfaction. Despite its promise, 3D printing still faces challenges such as limited biomimicry, insufficient bioinks, safety concerns, and weak regulatory oversight [8]. Without proper treatment, bone and joint injuries can worsen, leading to infections, tissue necrosis, and loss of function, while current solutions like grafts and metal implants have significant limitations, including immune rejection and short lifespans. Hydrogel-based 3D printing has emerged as a promising alternative, offering flexibility, biocompatibility, and repair capabilities, with wide material variety and potential in applications like tissue repair, infection control, and cancer treatment. Hydrogels, which form water-rich, cross-linked polymer networks, are ideal for 3D bioprinting due to their ability to retain shape while allowing nutrient and solute diffusion, making them a key material in next-generation orthopedic treatments [9]. Three-dimensional printing materials, such as Ti-6Al-4V used in selective laser melting (SLM), allow for the creation of personalized, anatomically accurate medical implants. However, the rougher surface topography of raw 3D printed implants can increase the risk of bacterial colonization and biofilm formation compared to smoother, coated commercial alternatives. While offering great customization potential, 3D printing materials still require refinement to balance biocompatibility, mechanical performance, and resistance to infection [10]. The most common applications of 3D printing in medicine are shown in Figure 1.

The purpose of this review paper is to present the current state of knowledge on the applications of 3D printing technology in orthopedic surgery and musculoskeletal traumatology, with a particular focus on its impact on improving surgical precision, personalizing treatment, and reducing patient recovery time. This paper aims to demonstrate the role of 3D printing not only as a tool to support clinical practice by creating anatomical models, implants, and surgical instruments tailored to individual patient needs but also as an innovative didactic tool in medical education. In addition, the analysis covers the potential development of this technology in the context of personalized medicine and possible directions for its further implementation in orthopedics and related medical fields.

## 2. Summary of 3D Printing Technology

The first step in creating 3D models is acquiring high-quality images using techniques such as CT and MRI scans. The resolution, accuracy, and quality of the 3D reconstruction depend directly on the quality of the 2D images acquired. The two most commonly used imaging modalities are multi-row computed tomography (MDCT) and magnetic resonance imaging (MRI). MDCT is preferred in orthopedics due to its high contrast and ability to produce thin axial images with a cross-sectional thickness of less than 1 mm. These images are characterized by isotropic voxels, making them ideal for high-resolution 3D post-processing. Although MRI allows better visualization of soft tissues, such as articular cartilage, the quality of the resulting images is inferior due to longer scan times and MRI’s sensitivity to motion artifacts. Often, MRI is used to supplement images taken by CT. The acquired images are stored in DICOM (Digital Imaging and Communications in Medicine) format, which standardizes the storage, exchange, and transmission of data. Postprocessing software uses these files to reconstruct the data. Techniques such as multiplanar reformation (MPR) allow for the creation of non-axial images, which facilitate clinical interpretation. For example, this is useful in cases where a fracture is not visible on an axial section. Other visualization methods, such as volume rendering, provide comprehensive 3D views of the dataset. Image segmentation using thresholding techniques allows for isolating regions of interest based on differences in voxel intensity. Once segmentation is complete, these data regions are used to generate 3D objects. These models are then converted into computer-aided design (CAD) representations. They consist of triangular polygons. The resolution of the model depends on the number of polygons used. Finally, the CAD data are exported in STL (stereolithography) format for 3D printing [9].

Not surprisingly, 3D printing technology continues to gain prominence, particularly in biomedical engineering. The products derived from this method can accurately replicate various complex structures while being produced with relatively low production effort and high personalization based on the patient’s anatomy. The main 3D printing technologies include vat photopolymerization, material extrusion, direct ink writing, powder bed fusion, and sheet lamination [11]. These additive manufacturing techniques along with their subtypes, working mechanisms, and advantages are briefly summarized in the table below (Table 1).

Additive manufacturing has introduced numerous possibilities in orthopedics, including applications such as high-precision anatomical models for preoperative planning, patient-specific 3D printed fixation plates, porous bone scaffolds, and custom metal implants [2]. For example, powder bed fusion is an excellent technology for fabricating biomedical implants, often load-bearing, due to the possibility of using metals such as titanium alloy or stainless steel [17]. On the other hand, direct ink writing is particularly useful for printing with soft, biocompatible materials like hydrogels, making it suitable for the fabrication of tissue constructs such as cartilage, bone, or skin [14]. Because the process does not involve heat, it allows the use of natural hydrogels, such as cellulose, chitosan, alginate, hyaluronic acid, gelatin, and collagen, which are advantageous due to their biodegradability and biocompatibility. DIW is an important technique in regenerative medicine, as hydrogels might be used in wound management, stimulating the healing process, as well as in controlled drug delivery and tissue engineering [14]. Meanwhile, vat photopolymerization is widely used in dentistry to fabricate implants, crowns, and veneers and also in orthopedics to produce various bone scaffolds, bone grafts, and even in tissue-engineering constructs [12]. Nevertheless, each of these technologies has certain limitations, including high cost, significant energy consumption, long printing time, and possible suboptimal surface finish or poor mechanical properties [18]. Additive manufacturing offers transformative possibilities in medicine and orthopedics; nonetheless, further research on these methods is needed in order to enhance accessibility for clinical application in hospitals and clinics.

## 3. Clinical Applications in Orthopedic Surgery

The clinical implementation of 3D printing in orthopedic surgery has expanded rapidly in recent years, offering tailored, efficient, and often more effective solutions across a wide spectrum of musculoskeletal conditions. This section reviews the most prominent applications, ranging from patient-specific implants to preoperative planning and complex oncologic reconstructions.

### 3.1. Patient-Specific Implants and Prostheses

The integration of 3D printing in orthopedic surgery has enabled the design and fabrication of PSIs and prostheses that are tailored to an individual’s unique anatomy and pathology. This personalized approach provides significant clinical benefits in terms of fit, biomechanical performance, and long-term outcomes. One of the most promising fields for 3D-printed PSIs is hip and pelvic surgery. Custom-made implants, particularly in cases involving severe acetabular bone loss or pelvic tumors, offer a better anatomical match than standardized components. By using CT-based data, implants can be produced with optimized surface geometry for osseointegration, including trabecular structures fabricated via SLM to improve biocompatibility and mechanical strength. These implants can significantly enhance stability, reduce surgical time, and improve functional recovery [19]. Similarly, in hip and knee arthroplasty, PSIs have been used to optimize implant positioning and improve surgical accuracy. Customized implants such as acetabular cups, femoral stems, tibial base plates, and cutting guides allow for better anatomical reconstruction, especially in revision surgeries or patients with bone defects. While outcomes are generally favorable, the current literature highlights the need for further studies to determine cost-effectiveness and long-term durability [20]. In wrist surgery, particularly for irreparable scaphoid damage, patient-specific scaphoid prostheses designed from CT scans of the contralateral side are becoming a viable salvage option. These implants help restore carpal kinematics and prevent collapse, and their success is highly dependent on precise ligament reconstruction and biomechanical fit [21]. In the field of cranioplasty, in-house fabrication of PSIs using 3D printing has proven to be not only effective but also cost-efficient. A case series involving 31 patients undergoing patient-specific 3D printer-assisted cranioplasty showed good functional and cosmetic outcomes, with a mean satisfaction score of 7.8/10. The implants were created using a desktop 3D printer and polylactic acid templates, allowing for rapid and affordable production directly at the point of care [22]. Innovative concepts such as modular, LEGO^®^-inspired titanium scaffolds represent the next evolution in patient-specific solutions. These SLM-printed, stackable scaffold units can be assembled intraoperatively to match complex defect geometries. They demonstrate biocompatibility, osteogenic potential, and sufficient mechanical integrity for use in bone defect reconstruction, while also offering cost and logistical advantages for application in resource-limited settings [23].

### 3.2. Preoperative Planning and Surgical Guides

Preoperative planning is essential for ensuring optimal outcomes in orthopedic and craniofacial surgery. Traditional methods relying on two-dimensional (2D) imaging and surgical experience often fail to capture the complexity of anatomical structures, which can lead to suboptimal intraoperative decisions. Three-dimensional printing offers a transformative solution, enabling the creation of tangible, patient-specific models that enhance anatomical visualization, improve planning accuracy, and support better communication with the surgical team and the patient [24,25]. The impact of this technology is supported by evidence: a systematic review found that 82% of studies on 3D printing and preoperative planning reported improved surgical outcomes when 3D-printed models were used instead of standard preoperative methods [25]. Three-dimensional printing allows surgeons to convert medical imaging data—most commonly from CT or MRI—into highly detailed physical models. This process involves three main steps: image acquisition, segmentation and postprocessing, and printing. High-resolution input data and accurate segmentation are key to producing reliable surgical models and guides. Technologies such as stereolithography (SLA) or material extrusion FDM can be chosen depending on the desired resolution, cost, and materials [25]. Beyond anatomical models, 3D printing enables the fabrication of patient-specific surgical guides and fixation hardware. These custom tools, designed using CAD software, have been shown to significantly improve the accuracy and efficiency of surgical procedures. This technique is beneficial not only in orthopedics but also presents a valuable method for various types of surgeries, including orthognathic surgery. In a prospective study involving 18 patients undergoing advancement genioplasty, it was demonstrated that in-house-designed 3D-printed surgical guides and fixation plates could transfer the virtual surgical plan to the operating field with a median deviation of just 0.19 mm. The local deviations at the Menton (Me—the most inferior point on the mandibular symphysis in the midline) and Pogonion (Pog—the most anterior point on the chin in the midline) landmarks were 0.67 mm and 0.41 mm, respectively, and no postoperative complications were reported within the 6-month follow-up period. These results support the high precision and clinical safety of custom 3D-printed guides in accurately transferring preoperative planning to surgical execution [26]. Moreover, preoperative planning plays a crucial role in the selection of the appropriate bone grafting method, taking into account factors such as the size and location of the defect, the patient’s condition, and the biological and mechanical properties required for successful integration [27]. In a 2018 study, a virtual prototype of a bipolar hip joint endoprosthesis was developed using Solid Edge ST8 software, comprising anatomically inspired components such as a femoral shaft, modular head, and a dual- spherical socket system designed for precise anatomical fit. Finite element analysis of the assembled model demonstrated that post-implantation stress was predominantly concentrated in the femoral neck region, with significantly increased pelvic stress observed in cases simulating cartilage degradation [28].

### 3.3. Custom-Made Orthotics and External Devices

Three-dimensional printing is revolutionizing the design and fabrication of orthotic devices and external assistive technologies by enabling fast, cost-effective, and highly individualized production. Traditional orthosis manufacturing methods often require labor-intensive, time-consuming manual shaping and thermoforming processes. In contrast, 3D printing offers precise replication of anatomical structures based on digital scans, allowing for custom-fit designs with complex geometries that are difficult to achieve using conventional techniques. A comprehensive narrative review by Choo et al. analyzed 22 clinical studies and demonstrated that 3D-printed orthoses were comparable or superior to conventional ones in terms of biomechanical performance, kinematics, and patient-reported outcomes such as comfort and satisfaction. Notably, orthoses created through 3D printing were shown to reduce pain, improve function, and increase compliance due to their lighter weight, breathability, and more ergonomic fit [29]. The technology is being widely applied in upper and lower limb orthoses (e.g., wrist splints, ankle–foot orthoses, custom insoles) as well as spinal orthoses, particularly in patients with adolescent idiopathic scoliosis. In this domain, a recent systematic review by Beygi and Wong found that 3D-printed spinal braces achieved outcomes comparable to or better than conventional braces in terms of Cobb angle correction, user satisfaction, and clinical safety. Importantly, the studies included in this review also highlighted reduced lead times and improved customization possibilities, making 3D-printed spinal orthoses an increasingly attractive option in clinical practice [30]. Moreover, advances in mobile 3D scanning and CAD software have enabled clinicians to digitize patient anatomy using accessible devices like iPads and iPhones. This promotes the scalability of custom orthotics manufacturing across various clinical settings and improves reproducibility. Additionally, digital files can be easily stored and reused for reprinting in the case of device damage or loss [31]. Gutierrez et al. further emphasized that the adoption of 3D printing has significantly shortened the time from patient evaluation to prosthesis delivery, especially in diagnostic socket production, while allowing clinicians to focus more on patient care by reducing labor-intensive fabrication steps [32]. Beyond orthotics and implants, 3D printing is increasingly used to produce customized intraoperative tools and surgical instruments. These include cutting guides, drilling templates, navigation jigs, fixation systems, and instrument handles that conform precisely to patient-specific anatomy. According to Cornejo et al., 3D-printed surgical guides have been shown to enhance precision during resections and osteotomies, especially in comparison to freehand techniques, reducing operative time and improving outcomes [33]. The technology enables the rapid prototyping of surgical instruments, which can be sterilized and used directly in the operating room, particularly in resource-limited or remote environments where conventional supply chains may be disrupted.

### 3.4. Spine Surgery

Spine surgery has emerged as one of the most dynamic fields for the application of 3D printing technology in orthopedics. The most common clinical uses include patient-specific drill guides, anatomical models, and customized implants, which are designed to improve surgical precision and reduce complication rates. A large-scale systematic review covering over 100 studies and 2000 patients showed that 3D-printed drill guides significantly improved the accuracy of pedicle screw placement and reduced operative time in 88% of cases, although some procedures became longer due to technical complexity [34]. Additionally, titanium implants produced with technologies like SLM and electron beam melting (EBM) have been successfully used for spinal reconstructions, especially in oncology and complex deformity cases. A scoping review of randomized controlled trials further confirmed that 3D printing leads to statistically significant improvements in operative time, blood loss, pain reduction, fluoroscopy use, and functional outcomes, without increasing the risk of complications [35]. These findings support the clinical relevance of 3D printing in both preoperative planning and intraoperative guidance. Moreover, a recent retrospective case–control study by Pan et al. demonstrated that the use of full-scale 3D-printed spine models in severe spinal deformity correction (e.g., kyphoscoliosis) not only increased the rate of accurate pedicle screw placement (72.5% vs. 69.0%) and reduced blood loss but also allowed surgeons to confidently perform more advanced osteotomies (three-column: 85.7% vs. 60%) [36]. In recent years, biomaterials research has introduced synthetic bone graft substitutes such as HA-DBM composites and hyperelastic bone^®^, which can be 3D printed to enhance spinal fusion without requiring recombinant growth factors. Moreover, novel peptide-based materials like peptide amphiphile hydrogels (PAHs) offer bioactive and customizable scaffolds for tissue engineering in spinal applications [37]. Despite the promising results, barriers such as high costs, technical complexity, and a need for further high-quality evidence still limit widespread clinical adoption. Nevertheless, ongoing development in imaging, materials science, and printing methods is expected to overcome these limitations and further establish 3D printing as a transformative tool in spine surgery.

### 3.5. Oncologic Orthopedic Surgery

Three-dimensional printing has emerged as a transformative tool in orthopedic oncology, addressing major challenges in preoperative planning, tumor resection, and complex skeletal reconstruction. It allows for the creation of PSIs and custom implants, tailored precisely to individual anatomical and oncological needs. In a comprehensive narrative review, Spałek et al. [38] highlighted how 3D printing supports limb-sparing procedures by facilitating precise resections and reconstructions, particularly in anatomically complex or previously non-reconstructable cases. The authors stressed the relevance of 3D printing not only in orthopedics but also in thoracic, craniofacial, and pelvic tumor surgery. Reported benefits include improved functional outcomes, shorter recovery times, and enhanced quality of life when compared to amputation or traditional reconstruction methods. Kotrych et al. [39] emphasized the advantages of custom-made 3D-printed implants in musculoskeletal oncology, particularly for pelvic and sacral tumor resections. Using technologies such as EBM and SLM with titanium alloys, implants can be fabricated with porous structures that promote osseointegration and reduce stress shielding. These implants are often used in combination with preoperative 3D models and osteotomy guides, resulting in high anatomical accuracy and fewer complications. The integration of navigation systems and 3D-printed tools has further improved intraoperative precision. In a study by McCulloch et al. [40], the use of navigation-assisted resection combined with PSIs enabled surgeons to achieve optimal oncological margins while minimizing trauma to surrounding tissues. This hybrid approach is particularly valuable in the management of bone sarcomas located in regions like the pelvis and scapula, where spatial orientation is critical and surgical exposure is limited. The benefits of 3D printing extend beyond orthopedic oncology. In head and neck cancer surgery, CAD/CAM technology and 3D printing have advanced the field of microvascular bone reconstruction, especially for mandibles and maxilla. Virtual planning, custom osteotomy guides, and pre-bent titanium plates have significantly reduced ischemia time, increased precision, and enabled dental rehabilitation integration during primary procedures [41]. These workflows are increasingly being adopted in major oncologic centers, with ongoing research into bioprinting and integrated therapeutic implants.

In the following table (Table 2), we briefly summarize the patient-specific applications of 3D printing in orthopedic surgery presented in selected studies.

## 4. Applications in Traumatology and Fracture Management

Traumatology is a unique and complex orthopedy branch because of diverse types of fractures, which often require individual approaches. In this regard, 3D printing seems to be an extremely useful method, as there are several areas in which it might be applied. Therefore, this section will present some advantages of using 3D printing technology in traumatology, based on insights from selected studies outlined below.

### 4.1. Upper Limbs

The first area of application is fractures including bones in which morphology varies considerably among different patients. The first example is the clavicle, whose fractures are common and might require operative fixation with the use of plates. Three-dimensional printing technology to make detailed preoperative plans by creating specific anatomical models was proven to raise the efficacy of inexperienced surgeons in opposition to relying exclusively on CT scans [42]. Additionally, it has significantly reduced the number of discomfort-related secondary surgeries, due to the improper adjustment of plates [43]. As a second example, studies involving the humerus showed that using 3D-printed models contributed to significantly reducing the operating time, decreasing intraoperative blood loss, and lowering fluoroscopy time in comparison to conventional methods [44,45]. Furthermore, a retrospective cohort 2024 study on 51 patients observed that the 3D printing group not only had reduced surgery time but also had higher implant placement accuracy, a lower prevalence of heterotopic ossification, and smaller pain scores than the traditional surgery group [46].

### 4.2. Acetabulum and Pelvis

Three-dimensional printing is an excellent method for visualizing complex structures, especially those located in areas hard to access. Not only are acetabular fractures, for example, due to their deep location, difficult to access, but the intraoperative selection of fittable plates is also associated with longer operating time and increased patient morbidity [47]. A prospective randomized case–control study by Maini et al. applied the 3D reconstruction of patients’ acetabula to preoperatively contour plates of the case group, oppositely to the control group with plates contoured intraoperatively. Blood loss and surgical time were lower in the study group, along with better postoperative reduction [48]. Closed reduction is typically preferred over open reduction, mainly due to its minimally invasive nature, leading to smaller tissue damage, lower blood loss, and fewer complications. When it comes to unstable pelvic fractures, the complexity of the area’s anatomy poses considerable difficulty in precisely inserting screws in order to achieve proper fixation, without damaging surrounding nerves and vessels [49]. In a retrospective observational study by Cai et al. [49], patients with unstable pelvic fractures were divided into study and control groups. The study group, consisting of 65 patients, had 3D-printed models of their pelvises, subsequently used to simulate the operation using minimally invasive cannulated screw treatment. For the control group, involving 72 patients, the surgical procedures and fixation method were the same, but the operation plan was performed using conventional X-ray film and 2D and 3D computed tomography data. In the 3D printing group, the duration of surgery and time of fluoroscopies were lower than they were in the control group, yet there was no significant difference when function outcomes were taken into consideration. Interestingly, a retrospective study by Hung et al. [50] on total of 167 participants, aiming to assess whether the presence of 3D-printed models for male nongeriatric patients with pelvis or acetabular fractures would shorten the length of hospital stay (LHS), discovered no such association. A different retrospective study, conducted on 76 older adults (aged ≥60 years) with the same type of fractures, correspondingly did not find a significant difference in hospital length of stay for patients who had 3D models printed [50].

### 4.3. Lower Limbs

As complex tibial plateau fractures are difficult to manage, a study by Duan et al. [51] retrospectively analyzed 22 patients treated with customized plates using 3D printing technology. In comparison to the control group, also consisting of 22 patients, which was treated with traditional plates, the experimental group experienced shorter surgical time, lower intraoperative blood loss, and a number of fluoroscopies, and it presented better knee joint flexion angles and better knee joint HSS scores. On the contrary, the control group required shorter preoperative preparation and LHS. Another example of combining 3D printing technology with personalized custom-made steel plates is a retrospective analysis by Liang et al. [52], which utilized it to treat complex distal intra-articular fractures of the trimalleolar ankle. The results of this study, which was conducted on a total of 48 patients, divided equally into two groups, included a higher rate of successful fracture reduction, a smaller incidence of complications, and superior ankle joint function in group with personalized plates. Therefore, customized plates derived with the use of 3D models might result in better joint function, which can be particularly beneficial to young, active patients.

### 4.4. Ligament Reconstruction

When it comes to structures as complicated as the knee, surgeons require extreme precision to achieve satisfactory results. This is an area where 3D printing technology demonstrates significant potential. A randomized experimental study performed on 10 cadaveric knees reported that in knee ligament reconstruction, the accuracy of mean angular deviation was significantly greater with the use of 3D-printed, patient-specific instrumentation, in comparison with the freehand technique performed by experienced surgeons [53]. Furthermore, an experimental study by Liu et al. [54] involved using surgical implants of the anterior cruciate ligament (ACL) created by desktop 3D printers, in a rabbit model. The effectiveness of ACL reconstruction using a tendon graft depends on proper healing within the bone tunnel. In this research, a 3D-printed porous scaffold made from PLA filament was coated with hydroxyapatite and seeded with mesenchymal stem cells (MSCs), due to their potential for proliferation and collagen production. Afterward, implants were used to reconstruct rabbits’ anterior cruciate ligaments. Evaluation 12 weeks after the procedure showed that “the MSC-treated group achieved ideal bone-tendon healing compared to the other groups”. This research highlights the enormous potential of 3D-printed grafts to revolutionize ligament reconstruction procedures.

## 5. Benefits and Limitations

### 5.1. Benefits

Three-dimensional printing has been identified as having many useful applications, especially in complex orthopedic surgery cases. It facilitates a more comprehensive understanding of 3D anatomy, enabling more precise surgical planning. Its application holds great potential for enhancing patient care standards [55]. In addition, the advent of contemporary three-dimensional imaging and modeling software has enabled the generation of personalized designs that are compatible with the anatomy of patients and their needs [56]. This is especially important for patients with deformities and anatomical changes, such as bone tumors. The ability to shape implants using 3D technology ensures a precise fit in these patients which would be impossible with conventional implants [57]. Studies also show that the use of 3D printing is associated with reduced surgery time, less patient blood loss during surgery, and shorter fluoroscopy times. Of particular importance here is the use of 3D printing in preoperative planning [58]. Another advantage of 3D printing in orthopedic surgery is osseointegration. Printed implants can have a rough, porous structure that allows the bone to fuse directly with the intramedullary implant. This is not possible with most conventional implants. In addition, the mechanical properties characteristic of cancellous and cortical bone can be reproduced for the implant by using the appropriate porosity of the implant [7]. Moreover, current technology allows the use of modern materials in 3D printing technology. Hybrids of natural materials such as alginate, collagen, or hyaluronic acid with polymer-based synthetic materials are used. This allows the potential of each material to be exploited. As a result, these materials have the good mechanical strength and machinability of synthetics, while retaining the properties of natural materials, such as better biological performance in terms of cell differentiation [59].

### 5.2. Limitations

Along with the many benefits of 3D printing, there are also challenges. One of these is the time-consuming process of creating appropriately sized 3D models. This can sometimes take several days, preventing the use of this technology in trauma emergencies. Another challenge is the need for high-quality materials, which are often expensive. The time-consuming and costly nature of the process limits its widespread use [56]. Another limitation associated with 3D printing is regulatory challenges. These relate to 3D-printed structures containing living cells and bioactive materials. Various aspects are considered, such as the not fully understood long-term effects of bioprinting on patients’ bodies or the dependence of the functioning of mechanisms in tissue structures on many components, which make it difficult to meet the definition of performance included in regulations. In addition, there is currently a lack of standardization in 3D bioprinting technology. This applies to materials such as the cells and bioprints used, as well as the process itself. At the moment, standardization only concerns the terminology used in production and the guidelines created for the companies that manufacture the printers. The introduction of standardization would make it possible to optimize the process and ensure the safety and reproducibility of the manufactured product [57]. Implants made using 3D printing technology may carry a risk of postoperative infection. According to some studies, the risk of such infections is higher with 3D bioprinting than with conventional prostheses. This is due to the greater porosity of 3D implants, which provides a favorable environment for the development of micro-organisms, leading to the formation of a biofilm that can eventually lead to infection [60]. Another limitation that may hinder the widespread adoption of 3D printing technology for implant manufacturing is the need for properly trained personnel. Employees need to be familiar with medical modeling software. In addition, the implants produced must be characterized by an individualized fit for the patient. This requires additional staff training to transfer clinical data to digital models and produce personalized prostheses [60]. One potential solution to this problem would be to entrust the creation of suitable 3D models and their printing to third-party companies offering such services, which a clinic could work closely with.

## 6. Future Perspectives

Three-dimensional printing technology is constantly evolving and could have applications in many areas of medicine in the future. It offers the potential to create an unlimited number of 3D structures from a variety of materials, including living cells [61].

There is also a belief that 3D printing technology will be developed into 4D printing. Future materials would have the ability to adapt their properties to the external environment and parameters such as temperature, pH, and magnetic and electric fields. Bioprinting cells reorganize over time and produce new tissue. In contrast, biodegradable implant components degrade over time. This requires a thorough understanding of the self-organization of the biomaterials used, tissue healing, and the integration of printed designs. The complexity of this process requires many studies, both in vitro and in vivo [9,57]. In addition to its benefits for clinical practice, the use of 3D printing can enrich other areas of medicine, such as education. Three-dimensional printed models can be used in the training of students and junior doctors, providing an additional opportunity to enhance knowledge and skills alongside intraoperative and cadaveric learning [58]. It is reasonable to hypothesize that, in the future, 3D printing technology will be incorporated into clinical guidelines. Nevertheless, the fundamental factors in determining the feasibility of this endeavor will be the existence of substantial research supporting its widespread use and a reduction in the cost of 3D printing [59].

## 7. Conclusions

Three-dimensional printing represents a significant advancement across various medical specialties, particularly in orthopedics and traumatology. Its applications range from fracture management, customized implants, and prosthetic devices to preoperative planning and the fabrication of patient-specific surgical instruments. Additionally, 3D printing plays a valuable role in medical education through the creation of anatomical teaching models. Collectively, these innovations contribute to enhanced surgical precision, improved patient outcomes, and more effective training for healthcare professionals. The integration of 3D printing technology in orthopedic surgery marks a transformative advancement in personalized medicine, offering solutions tailored to each patient’s unique anatomy and clinical condition. Its applications span a wide array of surgical domains, from complex hip and pelvic reconstructions to spinal corrections, oncologic resections, and trauma care. PSIs and surgical guides fabricated using 3D printing have consistently demonstrated improved anatomical fit, enhanced biomechanical performance, reduced operative time, and higher surgical precision. These advantages are particularly evident in challenging clinical scenarios, such as acetabular defects, scaphoid reconstruction, or osteosarcoma resections, where conventional implants may fall short. In preoperative planning, 3D-printed models greatly enhance anatomical visualization and interdisciplinary communication, contributing to more accurate surgical execution and better outcomes. Similarly, in the design of orthotic and assistive devices, 3D printing allows for rapid, cost-effective customization, leading to improved patient comfort, satisfaction, and functional recovery. The growing body of evidence highlights the versatility and clinical utility of 3D printing across orthopedic subspecialties. In traumatology, it has improved the precision of fracture management, particularly in anatomically complex regions such as the pelvis, tibial plateau, and clavicle. In ligament reconstruction, 3D-printed guides and biologically enhanced scaffolds show promising results for improving graft integration and healing. Despite its numerous benefits, the widespread clinical adoption of 3D printing faces challenges, including cost-effectiveness, regulatory considerations, and the standardization of manufacturing protocols. Long-term outcome data and larger comparative studies are needed to fully validate its efficacy and justify routine use in clinical settings. Nonetheless, as the technology continues to evolve and become more accessible, 3D printing is poised to become an integral part of modern orthopedic and reconstructive surgery, driving innovations in patient care, surgical precision, and functional outcomes.

The integration of 3D printing into orthopedic surgery clearly marks a turning point in personalized patient care. Its ability to enhance surgical precision, reduce operative risks, and offer tailored solutions makes it a valuable addition to modern medical practice. However, despite its promise, the widespread adoption of this technology is currently hindered by practical and regulatory challenges, such as production time, material costs, and the need for specialized training. Looking ahead, continued technological advancements and improvements in standardization are expected to overcome many of these limitations. As research expands and costs decrease, 3D printing is likely to become more accessible, not only in clinical applications but also in education and research. The eventual shift towards 4D printing further underscores the dynamic future of this field, hinting at a new era where implants and structures will be capable of adapting to the body’s changing environment. In conclusion, while 3D printing in orthopedics is still evolving, its current contributions and future potential make it a revolutionary force in medicine—one that could redefine both treatment standards and educational practices in the years to come.

## Figures and Tables

**Table 1:** Summary of additive manufacturing techniques.

3D Printing Technology	Subtypes	Working Principle	Advantages	References
Vat Photopolymerization	stereolithography, digital light projector, liquid crystal display, two-photon polymerization	liquid resin monomers or oligomers are polymerized upon exposure to a specific light source	- high printing speed - precision in printing high-resolution structures	[12]
Material Extrusion	fused deposition modeling (FDM), direct ink writing (DIW), pellet extrusion, paste extrusion	FDM uses thermoplastic or viscoelastic materials, extruded through a nozzle and deposited into layers onto a substrate, using a heated printhead	low cost and energy consumption, high spatial resolution, ability to produce complex constructs	[13,14]
Direct Ink Writing	constitutes subtype of material extrusion technology itself	the working principle is similar to that of FDM, but the extrusion is pneumatic or mechanical, at a mild temperature	high accuracy and spatial resolution, possibility to use hydrogel materials	[14]
Powder Bed Fusion	selective heat sintering, selective laser sintering, selective laser melting, electron beam melting, multi-jet fusion	selectively fuses each layer of powdered material using a heat source (laser or an electron beam)	diverse range of possible materials, precision, ability to handle complex geometries	[15]
Sheet Lamination	laminated object manufacturing (LOM), ultrasonic additive manufacturing (UAM), plastic sheet lamination (PSL), laser-induced graphene (LIG)	bonds successive material layers using heat and adhesive to form a consolidated structure	low cost, fast manufacturing of large elements	[16]

**Table 2:** Comparison of selected studies on the application of 3D printing technology in patient-specific orthopedics.

Clinical Area	Application	Key Outcomes	Technologies Used/Materials	References
Preoperative Planning	3D models for surgical planning	82% of studies report improved surgical outcomes	SLA, FDM, CT/MRI-based modeling	[25]
Craniofacial Surgery	3D-printed surgical guides for genioplasty	No complications at 6 months	CAD-designed guides, in-house printing	[26]
Orthotics	3D-printed orthoses	Superior or equal biomechanical/kinematic outcomes; improved comfort, reduced pain, and increased compliance	3D scanning and CAD, mobile scanning, digital design	[29]
Scoliosis/Spinal Orthoses	3D-printed spinal braces	Comparable or better Cobb angle correction; reduced lead time; high customization	Stratasys machine and FDM technique/nylon (PA12)	[30]
Prosthetics	Diagnostic sockets and custom orthoses	Shorter production time; focus shifted to patient care	FDM, CAD/CAM technology/polylactic acid and Acrylonitrile Butadiene Styrene	[32]
Surgical Instruments	Cutting and drilling guides and navigation jigs	Increased precision in resections and osteotomies; reduced operative time	Sterilizable materials, rapid prototyping	[36]
Spine Surgery	Patient-specific drill guides and implants	Improved pedicle screw accuracy (88%); reduced time, pain, and blood loss	SLM, EBM/titanium	[34,35]
Severe Spinal Deformity	3D spine models for planning complex osteotomies	Higher screw accuracy (72.5%), more 3-column osteotomies (85.7%), and reduced blood loss	Full-scale spine models	[36]
Orthopedic Oncology	Tumor resection guides and custom implants	Better functional outcomes, osseointegration, and fewer complications	EBM, SLM, porous titanium implants	[38,39]
Head and Neck Oncology	Navigation-assisted resections and reconstruction	Optimal margins, minimized tissue trauma, and improved dental rehab integration	CAD/CAM technology, PSIs/ titanium guides/plates	[40]

## Abbreviations

The following abbreviations are used in this manuscript:

2D	two-dimensional
3D	three-dimensional
ACL	anterior cruciate ligament
AM	additive manufacturing
CAD	computer-aided design
CBL	case- based learning
CT	computer tomography
DICOM	Digital Imaging and Communications in Medicine
DIW	direct ink writing
EBM	electron beam melting
FDM	fused deposition modeling
LHS	length of hospital stay
LIG	laser-induced graphene
LOM	laminated object manufacturing
MDCT	multi-row computed tomography
MR	magnetic resonance
MSCs	mesenchymal stem cells
PAH	peptide amphiphile hydrogel
PSIs	patient-specific instruments
PSL	plastic sheet lamination
RP	rapid prototyping
SLA	stereolithography
SLM	selective laser melting
UAM	ultrasonic additive manufacturing
